# Microbial and Monosaccharide Composition of Biofilms Developing on Sandy Loams from an Aquifer Contaminated with Liquid Radioactive Waste

**DOI:** 10.3390/microorganisms12020275

**Published:** 2024-01-28

**Authors:** Tamara L. Babich, Nadezhda M. Popova, Diyana S. Sokolova, Andrei V. Perepelov, Alexey V. Safonov, Tamara N. Nazina

**Affiliations:** 1 Winogradsky Institute of Microbiology, Research Center of Biotechnology, Russian Academy of Sciences, Moscow 119071, Russia; microb101@yandex.ru (T.L.B.); sokolovadiyana@gmail.com (D.S.S.); 2 A.N. Frumkin Institute of Physical Chemistry and Electrochemistry, Russian Academy of Sciences, Moscow 119071, Russia; nm.popova.ipce.ras@gmail.com (N.M.P.); alexeysafonof@gmail.com (A.V.S.); 3 N.D. Zelinsky Institute of Organic Chemistry, Russian Academy of Sciences, Moscow 119991, Russia; andreivperepelov@gmail.com

**Keywords:** biofilms, aquifer, surface radioactive waste repository, high-throughput sequencing, 16S rRNA gene, denitrification, uranium reduction, biofilm matrix, scanning electron microscopy

## Abstract

The development of microbial biofilms increases the survival of microorganisms in the extreme conditions of ecosystems contaminated with components of liquid radioactive waste (LRW) and may contribute to the successful bioremediation of groundwater. The purpose of this work was to compare the composition of the microorganisms and the exopolysaccharide matrix of the biofilms formed on sandy loams collected at the aquifer from a clean zone and from a zone with nitrate and radionuclide contamination. The aquifer is polluted from the nearby surface repository for liquid radioactive waste (Russia). The phylogenetic diversity of prokaryotes forming biofilms on the sandy loams’ surface was determined during 100 days using high-throughput sequencing of the V4 region of the 16S rRNA genes. Scanning electron microscopy was used to study the development of microbial biofilms on the sandy loams. The ratio of proteins and carbohydrates in the biofilms changed in the course of their development, and the diversity of monosaccharides decreased, depending on the contamination of the sites from which the rocks were selected. The presence of pollution affects biofilm formation and EPS composition along with the dominant taxa of microorganisms and their activity. Biofilms establish a concentration gradient of the pollutant and allow the microorganisms involved to effectively participate in the reduction of nitrate and sulfate; they decrease the risk of nitrite accumulation during denitrification and suppress the migration of radionuclides. These biofilms can serve as an important barrier in underground water sources, preventing the spread of pollution. Pure cultures of microorganisms capable of forming a polysaccharide matrix and reducing nitrate, chromate, uranyl, and pertechnetate ions were isolated from the biofilms, which confirmed the possibility of their participation in the bioremediation of the aquifer from nonradioactive waste components and the decrease in the radionuclides’ migration.

## 1. Introduction

The operation of nuclear fuel cycle enterprises may result in the release of highly toxic and complex pollutants into the environment. The storage of waste during uranium ore mining and processing, as well as the preservation of spent nuclear fuel at radiochemical plants, pose significant environmental risks [1]. Of particular concern is contamination of the aquifers near radioactive waste repositories, such as the sludge and pulp storage facilities that were constructed in the middle of the 20th century [2,3]. These facilities contain highly saline solutions and brines in which mineral acids, heavy metals, and radioactive elements are present. Unfortunately, these hazardous substances seep through the storage facility walls and contaminate groundwater [4,5,6]. Over time, these pollutants can migrate through aquifers indefinitely, posing a threat to water sources and natural water bodies [7]. The high nitrate content in the waste also creates a favorable environment for the migration of long-lasting radioactive materials, such as uranium [5,8] and technetium [9], as well as the dissolved oxidized forms of redox-active metals like chromium, vanadium, and manganese. 

The penetration of pollutants into aquifers has a profound impact on the balance of electron donors and acceptors in underground ecosystems. This, in turn, triggers a series of biogeochemical reactions involving the iron, nitrogen, and sulfur cycles [10,11]. As a result, the presence of denitrifying and nitrate-reducing microorganisms leads to a decrease in nitrate levels in groundwater. This not only lowers the redox potential of the environment but also causes immobilization of the redox-active radionuclides in less soluble compounds [12,13]. Remarkably, microorganisms possess the ability to utilize various metals and metalloids such as technetium [14], uranium, and chromium [15], among others [16,17,18,19], in dissimilatory processes. 

Throughout the world, a profound wealth of knowledge has been amassed concerning utilization of the biogeochemical capabilities of the indigenous subsurface microorganisms. These abilities were proven to be instrumental in successfully mitigating the detrimental effects of nitrate pollution [20], uranium contamination [16,21,22,23,24], and the presence of technetium [25] as well as of other actinides [26] and heavy metal pollutants [27,28,29] within shallow aquifers. 

The success of an in situ bioremediation process hinges upon the unique characteristics of the microbial community, including its composition, activity, and stability against the harmful effects of heavy metals and radionuclides. The most favorable conditions for this process occur when microorganisms form biofilms on the surface of aquifer rocks [30,31,32]. These biofilms enable the microorganisms to adapt more efficiently to changes in the aquifer’s hydrodynamic regime, ensuring optimal pollutant removal even during groundwater movement. Furthermore, they facilitate efficient pollutant elimination via intricate biochemical reactions such as denitrification [33,34]. 

Biofilms are cell aggregates with complex functional connections enclosed in an exopolysaccharide (EPS) matrix [33]. This EPS matrix, accounting for a significant portion (50–90%) of the biofilm organic matter, is primarily composed of β-glucuronic acid [28]. The composition of monosaccharides within the matrix can vary considerably, with glucose, fructose, mannose, and arabinose being more prevalent, while rhamnose, ribose, xylose, and galactose are less commonly found. Additionally, the EPS matrix contains proteins (outer membrane proteins and proteins from mobile cell parts), lipopolysaccharides, and phospholipids [31,32,33,35]. These diverse components, including polysaccharides with carbonyl and amino groups, play a crucial role in the immobilization of metals like uranium within the biofilms, thereby hindering their mobility in the surrounding environment [23,36,37,38]. 

This study examined the microbial community in underground water with high levels of nitrate and radionuclide contamination that was collected from the upper aquifer near a preserved surface repository of liquid radioactive waste at the Siberian Chemical Combine (SCC, Tomsk region, Russia). Previously, experiments on in situ bioremediation of groundwater contaminated with liquid radioactive waste components have been conducted [23]. However, stimulation of the microbial community by a single injection of soluble organic compounds (e.g., electron donors) into the aquifer resulted in only temporary nitrate removal; the concentration of nitrates returned to the previous level within a few months due to the influx of contaminated water. We believe that the key to achieving a long-lasting purification effect in aquifers lies in the formation of stable microbial biofilms on the surface of sandy loams, which create a durable restorative-sorption barrier for nitrate ion removal and radionuclide immobilization. It should be noted that until the nitrates are removed from the groundwater, the radionuclides remain in an oxidized soluble form and cannot be reduced by bacteria and converted into an insoluble form. This makes the denitrification process particularly important as the first stage of remediation of groundwater from radioactive waste.

The objective of this study was to compare the composition of microorganisms and the exopolysaccharide matrix in biofilms formed on sandy loams that were collected from the aquifer in both the clean zone and the area with nitrate and radionuclide contamination. Additionally, from the biofilms were isolated bacteria capable of nitrate, chromate, uranyl, and pertechnetate reduction to determine their potential in bioremediation of the groundwater from nonradioactive waste components and the decrease in the metals’ and radionuclides’ migration.

## 2. Materials and Methods

### 2.1. The Object of the Study

The studied samples of underground water from the sandy loam aquifer horizon (depth of 10–15 m) were collected from observation wells W1 and W2, located in the area of the B2 preserved surface repository of liquid radioactive waste at the territory of the Siberian Chemical Combine (Seversk, Tomsk region, Russia) [23]. The composition and main parameters of the groundwater samples are presented in Tables S1 and S2. Observation well W1 is located outside the zone of groundwater contamination by liquid radioactive waste components. Observation well W2 is located in an area subjected to the technological impact of the reservoir. In addition to nitrate contamination, this well’s liquid also has an increased content of cesium, technetium, strontium, and uranium. Moreover, increased chromium content (0.56 mg/mL) was detected in the water from well W2.

Particle-size analysis of the sandy loam was determined using the standard method [39]. The particle size distribution of the sandy loam samples was characterized by predominance of the fraction of fine (0.25–0.01 mm) sandy particles (40% by volume) and a small fraction of dusty-clay particles (less than 10% by volume). The density of the solid soil particles was 2.67 g/cm^3^. The low value of the solid phase density results from the presence of organic matter, water-soluble salts, and low-density minerals (quartz, kaolinite) in the soil. X-ray structural analysis revealed quartz, albite, and chlorides, as well as a clay fraction (10–15% *v*/*v*) containing illites, smectites, and kaolinite in similar proportions. The elemental composition of the soil samples from the technologically modified aquifer horizon (W2) and the uncontaminated part of the formation (W1) is presented in Table S3. Apart from aluminum, silicon, and other elements that are part of clay aluminum silicates and quartz, the uncontaminated soil sample contained 2.75% by mass of iron oxide (III), and sulfur was present in trace amounts; the technogenic soil had increased levels of magnesium, calcium, and sulfur, possibly in the form of pyrite deposits.

### 2.2. Medium Composition and Cultivation Conditions

For isolation of the microorganisms, sandy loam samples (2 g each) from observation wells W1 and W2 were placed in hermetically sealed penicillin flasks and supplemented with liquid nutrient medium simulating the composition of groundwater. The gas phase was represented by air. The medium had the following composition (g/L of distilled water): NaHCO_3_—0.05; MgSO_4_·4H_2_O—0.04; CaCO_3_—0.02; NH_4_Cl—0.1; K_2_HPO_4_—0.2; KNO_3_—1.0; pH—6.8–7.0. Microelements were added to the medium (1 mL/L) [40]. Sodium acetate (2.0 g/L) as the organic substrate was added once at the beginning of the experiment. The medium with the soil was sterilized at 1 atm. Groundwater samples from wells W1 and W2 were added to the flasks as inocula (1 mL each). The microcosms were incubated for 365 days under stationary conditions at 20 °C. Thus, microcosms MW1 and MW2, containing both planktonic cells and biofilms, were obtained from wells W1 and W2. Samples for various analyses were taken after 1, 7, 12, 15, 20, 28, 40, 70, 100, and 365 days.

Pure bacterial cultures were isolated from dilutions of the microcosms via successive plating on the solid medium containing the following (per liter distilled water): bacteriological peptone—5.0; glucose—1.0; yeast extract—2.5; agar—15.0, pH—7.0–7.2. The purity of the strains was checked via microscopy of the colonies and 16S rRNA gene sequencing, as described in Section 2.7. Reduction of nitrates and heavy metals by the biofilm microbial community and by pure cultures was evaluated under anaerobic conditions using a nutrient medium with the following composition (g/L of distilled water): KH_2_PO_4_—0.75; K_2_HPO_4_—1.5; NaCl—0.8; Na_2_SO_4_—0.1; MgSO_4_·7H_2_O—0.8; KCl—0.1; acetate Na—2.0; pH—6.8–7.0. U^6+^, Cr^6^, Tc^7+^, and NO_3_^−^ were added as electron acceptors in the form of uranium nitrate, UO_2_(NO_3_)_2_ (50 mg/L); chromium oxide, CrO_3_ (30 mg/L); sodium pertechnetate, NaTcO_4_ (10 mg/L); and potassium nitrate, KNO_3_ (1.0 g/L), respectively. Argon was used as the gas phase in all cases. The pure cultures were incubated in media with nitrates and metals for 30 days under stationary conditions at 20 °C.

### 2.3. Analytical Methods

Elemental content in the water specimens was examined using the Thermo Scientific X Series2 Quadrupole ICP-MS (inductively coupled plasma mass spectrometer, Thermo Fisher Scientific, Waltham, MA, USA, https://static.thermoscientific.com/images/D01737~.pdf (accessed on 20 February 2023)) and the Thermo iCAP 6500 Duo ICP-AES spectrometer (Thermo Fisher Scientific, Waltham, MA, USA, https://fscimage.fishersci.com/images/D01567~.pdf (accessed on 20 February 2023)) right after collection and filtration (glass microfiber membrane, pore size 0.45 μm). The measurement of cation and anion concentrations was conducted using the Capel-105M CE system (LUMEX Instruments, Saint Petersburg, Russia, https://www.lumexinstruments.com/catalog/capillary-electrophoresis/capel-105m.php (accessed on 20 February 2023)). The determination of Eh and pH values was performed via an ANION-4100 pH meter/ionomer equipped with an Ag-AgCl reference electrode (Novosibirsk, Russia, http://www.anion.nsk.su/ (accessed on 20 February 2023)).

The radionuclide concentration was determined using gamma spectrometry with HPGe detectors (GEM-60195-P, ORTEC, Oak Ridge, TN, USA), alpha spectrometry with PIPS detectors (Alpha-Analyst, Canberra, Australia), and liquid scintillation spectrometry (TriCarb-2700TR, Canberra, Australia) after appropriate chemical separation procedures.

The X-ray diffraction (XRD) examination of the rocks’ composition was conducted at the Scientific Center of Shared Use, A.N. Frumkin Institute of Physical Chemistry and Electrochemistry, Russian Academy of Sciences, via an Aeris X-ray diffractometer (Malvern PANalytical, Malvern, UK) using CuKα radiation (40 kV, 15 mA). The samples, in a pre-dried state, were pulverized to a powder in a corundum mortar prior to XRD analysis. The subsequent preparation of the samples, clay minerals’ identification and elemental composition, and results interpretation were performed as described previously [12]. 

The biofilm organic carbon after biofilm formation was determined on the 100 V 2400 Series II CHNS elemental analyzer (Perkin Elmer, Waltham, MA, USA). The respiration activity of attached biofilms was identified using a 0.1% solution of 3-(4,5-dimethyl-2-thiazolyl)-2,5-diphenyl-2H-tetrazolium bromide (Dia-m, Moscow, Russia) or an MTT test [41,42,43]. Sugar analysis of the biofilms was made using GLC after full acid hydrolysis with 2 M CF_3_CO_2_H (120 °C, 2 h). Monosaccharides were identified through GLC of the alditol acetates on a Maestro 7820 GC (Interlab, Moscow, Russia) equipped with an HP-5ms column (0.32 mm × 30 m) using a temperature program of 160 (1 min) to 290 °C at 7 °C min^−1^. The absolute configurations of the monosaccharides were determined using GLC of the acetylated (S)-2-octyl glycosides as described [44]. The reduction of technetium and chromium was determined using diphenylcarbazide [45], while that of uranium was determined using arsenazo III, after liquid extraction of the reduced form with 1-(2-thenoyl)-3,3,3-trifluoroacetone (TTA) [46].

### 2.4. Microscopic Methods

Visualization of the microbial biofilms was conducted using a TESCAN VEGA-II XMU scanning electron microscope (TESCAN, Brno, Czech Republic). The composition and structure of the biofilms were analyzed using laser confocal scanning microscopy (Leica SP5, Leica, Wetzlar, Germany) and associated programs [47]. Samples were fixed with 96% alcohol and then rinsed with a phosphate-buffered saline. Subsequently, the samples were treated with wheat germ agglutinin (WGA) conjugated with AlexaFluor 488 (W11261 Thermo Fisher; 1:500 dilution). WGA binds to the monosaccharides of bacterial cell walls and extracellular polymeric substances (EPSs). The fluorescent dye SYTO 11 (S7573, Thermo Fisher, USA; 1:1000 dilution) binds to nucleic acids. Staining was conducted as described in [48]. To assess the protein component of the biofilms, a fluorescent derivative of fluorescein (FITC) was used (Merck, Darmstadt, Germany). The samples were washed three times with a phosphate buffer and analyzed using the Leica SP5 confocal microscope. An argon laser was used with a wavelength of 488 nm for WGA excitation, 514 nm for SYTO 11, and 543 nm for FITC detection. The Nomarski contrast was used to visualize unstained particles (sand) [49]. The acquired images were analyzed using the Imaris 7.0.0 software package (Bitplane, Zurich, Switzerland) and Comstat 2.1 software (ImageJ, USA). 

### 2.5. DNA Extraction from Pure Cultures and Biofilms

The biomass from the sandy loam surface in the microcosm was washed with sterile distilled water and preserved with ethanol (1:1 *v*/*v*). It was then filtered through membrane filters with a pore size of 0.22 µm (Millipore, Merck, Darmstadt, Germany). The biomass from the filter surface was washed with a solution containing 0.15 M NaCl and 0.1 M Na_2_EDTA (pH 8.0). For the extraction of genomic DNA from the biofilms, the Pure LinkTM Microbiome DNA Purification Kit (Thermo Fisher Scientific, Waltham, MA, USA) was used according to the manufacturer’s recommendations.

### 2.6. High-Throughput Sequencing of V4 Fragment of the 16S rRNA Genes

To determine the composition of the biofilm microbial communities, the hyper-variable V4 region of the 16S rRNA gene was amplified. Amplification was carried out in a mixture containing 5 μL of each primer (6 μM concentration), 5 μL of DNA solution, and 15 μL of PCR mix (1 U of polymerase, 0.2 mM of each dNTP, 2.5 mM Mg^2+^), using the primers Pro341F-Pro805R [50]. Each sample was amplified in triplicate, which were then combined and purified using electrophoresis on a 2% agarose gel using a PCR product extraction and purification kit (Eurogene, Moscow, Russia). Sequencing was performed using the MiSeq platform (Illumina, San Diego, CA, USA) and the MiSeq V3 reagent kit (600 cycles) (Illumina, San Diego, CA, USA) according to the manufacturer’s recommendations. Gene libraries were obtained using dual barcoding, as described earlier [51]. Assuming that the composition of the libraries corresponds to the composition of actual microbial communities, we present the composition of biofilms considering the quantity of 16S rRNA gene sequences in the libraries.

### 2.7. Identification of Pure Cultures

The DNA from pure cultures was isolated using the DIAtom^TM^DNAPrep100 reagent kit (Biokom, Moscow, Russia), following the manufacturer’s recommendations. The purified DNA extract was used as a template for PCR. The isolated DNA from pure cultures was used for polymerase chain reaction (PCR) with primers universal for representatives of the Bacteria domain, 8–27f [5′-AGAGTTTGATCCTGGCTCAG-3′] and 1492r [5′-GGTTACCTTGTTACGACTT-3′] [52,53]. PCR was performed in a reaction mixture (25 µL) containing 10–50 ng of DNA template on an iCycler thermocycler from BioRad (Hercules, CA, USA) using the following program: 1 cycle of 3 min at 94 °C followed by 30 cycles (0.5 min at 94 °C, 0.5 min at 50 °C, 0.5 min at 72 °C) and a final extension of 7 min at 72 °C. The length of the amplified fragments was evaluated on a 1.0% agarose gel stained with ethidium bromide. Sequencing of the PCR products of the 16S rRNA gene fragments was performed on an automatic sequencer, the 3730 DNA Analyzer, using the BigDye^TM^ Terminator v3.1 cycle sequencing kits (Applied Biosystems, Austin, TX, USA) according to the manufacturer’s instructions. The obtained nucleotide sequences were preliminarily analyzed using the online resource BLAST in the NCBI GenBank database. The obtained sequences were compared with reference organism sequences. Editing was performed using the BioEdit Sequence Alignment Editor software program (https://bioedit.software.informer.com/, v7.7, accessed on 25 May 2023) [54].

### 2.8. Bioinformatics Analysis

The sequenced right and left readings of the 16S rRNA gene fragments were combined using the SeqPrep program (https://github.com/jstjohn/SeqPrep, v1.3.2, accessed on 4 October 2016) [55]. Bacterial community libraries were created using the SILVA online resource (https://www.arb-silva.de/ngs/, r138.1, accessed on 14 March 2023) [56]. For the analysis of the library sequences, they were first merged into operational taxonomic units (OTUs) with a similarity level of 97%. The online resource ClustVis (https://biit.cs.ut.ee/clustvis/, accessed on 8 April 2022) [57] was used to create principal coordinate analysis (PCA) plots and heatmaps. The software iVikodak [58] was used to predict the functional characteristics of the bacterial communities. Venn diagrams were constructed using the online resource (http://bioinfogp.cnb.csic.es/tools/venny/, accessed on 12 April 2022) [59].

### 2.9. Nucleotide Sequence Accession Numbers

The obtained 16S rRNA gene sequences of pure cultures were deposited to GenBank under accession nos. MK567698, MK567783, and MK571249. The 16S rRNA gene fragment sequences of bacteria from biofilms are available in the NCBI SRA database under the following accession number: SUB14021502, BioProject PRJNA1054355.

## 3. Results

### 3.1. The Composition of Biofilms on Sandy Loams

**Figure 1:**
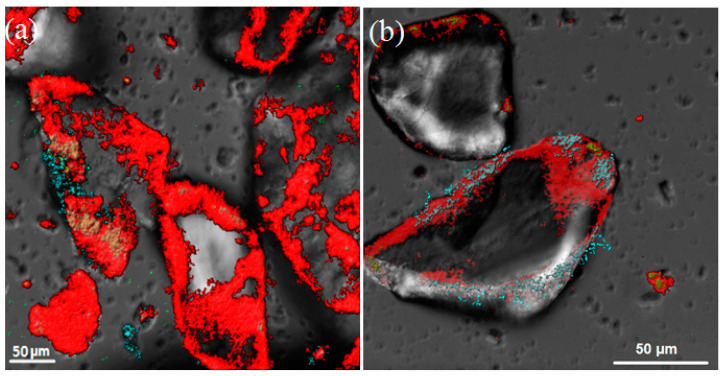
The biofilm composition on sandy loams using fluorescence imaging with a Leica SP5 confocal microscope (Leica, Wetzlar, Germany) at 400× magnification. The distribution of nucleic acids (green and beige), proteins (blue), and polysaccharides (red) in the biofilms of MW1 (**a**) and MW2 (**b**) on day 15 of formation is depicted.

**Figure 2:**
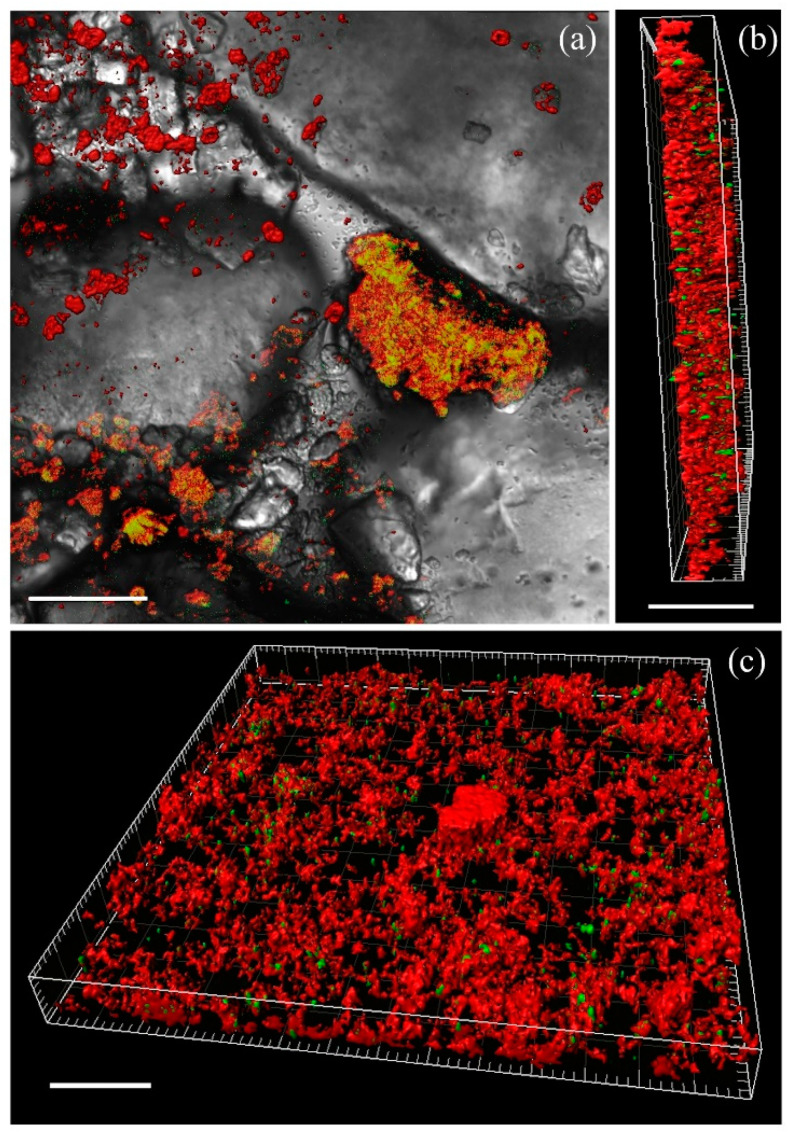
The MW1 biofilms’ structure on sandy loams on day 15 as detected with laser confocal scanning microscopy (Leica SP5 confocal microscope (Leica, Wetzlar, Germany), magnification 400×). The nucleic acids are stained green with SYTO 11 GREEN; the mono-, oligo-, and polysaccharides are stained red with WGA, the dyes overlapping gives a yellow color. Bar, 50 µm. (**a**) epilithic biofilm on sandy loam; (**b**) longitudinal section showing biofilm thickness; (**c**) frontal biofolm z-stack.

Microphotographs of the sandy loams’ surfaces with developing biofilms were obtained using confocal scanning microscopy. The analysis of the biofilms was conducted on day 15 of the experiment after inoculation of the medium with water samples containing sandy loams from wells W1 and W2 as mineral carriers. It was previously observed that maximum biomass accumulation on the solid carrier occurred after 14 days of biofilm formation [60]. Studying the microphotographs of the sandy surface (Figure 1 and Figure 2) revealed uneven distribution of both microbial cells and polysaccharides. 

Areas were identified where cell aggregates surrounded by a polysaccharide matrix predominated, as well as areas dominated by attached individual cells. Additionally, areas stained with FITC, which detects proteins, were observed outside the polysaccharide matrix. Such positioning may indicate the formation of biofilms on unoccupied areas of the sandy loams.

A stack analysis of the biofilms (Comstat 2.1 from ImageJ) enabled the determination of their average thickness for both samples, which ranged from 30 to 60 μm regardless of the degree of contamination by liquid radioactive waste components (Figure 2b).

On day 15 of the experiment, the MW1 biofilm from the uncontaminated water covered 40.3% of the sand surface area and contained 3.7% nucleic acids (NA), 24.0% proteins, and 72.3% polysaccharides as a percentage of the total biofilm area. The MW2 biofilm from the contaminated sample covered 31.9% of the entire soil surface and contained 2.8% NA, 22% protein, and 75.2% polysaccharides. Uneven development of the biofilms on the sandy loam samples may be attributed to their diverse mineral compositions and conditions for the physicochemical adhesion of microorganisms. It can be expected that the least biofilm growth will occur on quartz grains, while the microbial matrix will develop more on minerals containing biogenic elements (iron, potassium, calcium, sulfur), mica, iron-bearing minerals (hematites, biotites, pyrites), and feldspars, as well as on quartz grains covered with a clay film.

### 3.2. The Dynamics of Biofilms’ Formation on Sandy Loams

**Figure 3:**
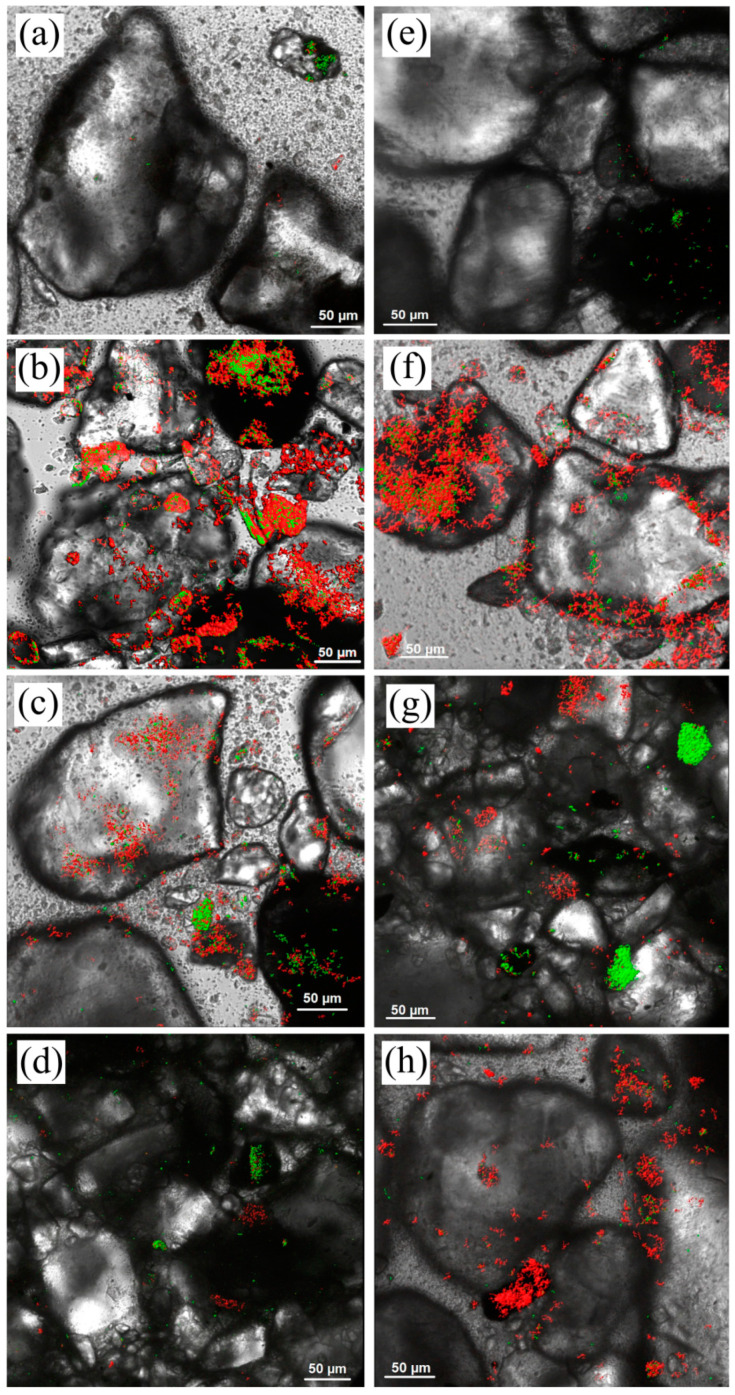
Images of biofilms on sandy loams from observation wells W1 (**a**–**d**) and W2 (**e**–**h**) at days 7, 15, 40, and 100 revealed with laser confocal scanning microscopy. Nucleic acids are stained green using SYTO 11 GREEN, while mono-, oligo-, and polysaccharides are stained red with WGA. Leica SP5 confocal microscope (Leica, Wetzlar, Germany), magnification 400×.

The multispecies biofilms’ formation dynamics on clayey–sandy rocks are illustrated with micrographs obtained using a laser confocal scanning microscope (Figure 3).

**Figure 4:**
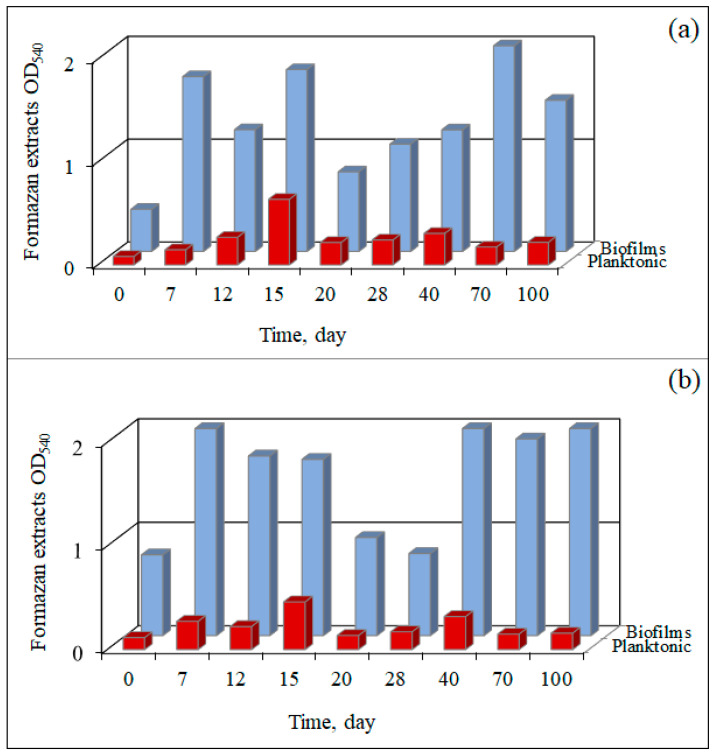
The respiratory activity (MTT test) of attached (biofilm) cells and planktonic cells in microcosms MW1 (**a**) and MW2 (**b**).

During the initial 24 h following inoculation of the liquid medium with the underground water microbial community, a gradual adhesion of planktonic microorganisms to the sand particles was observed. Rapid primary adhesion was also noted in a previous study [61]. From day 7 to day 15, there was intense formation of the EPS matrix in the biofilm. This period also exhibited the highest diversity of biofilm formations, primarily epilithic, tightly adhering to the rock surface (Figure 3b,f). Subsequently, there was a decrease in the amount of biofilm matrix, which may be attributed to cell lysis, dispersion of cells from the biofilms into the liquid medium followed by their reattachment to surfaces and formation of new colonies, or changes in the microbial community composition and a shift in the dominant members. In the microcosm MW2, the quantity of detected nucleic acids increased on day 40, correlating with the peak in respiratory activity of the attached microbial community (Figure 4). A similar increase in nucleic acids and respiratory activity was observed on day 70 in the microcosm MW1. Microscopic examination revealed that in both microcosms, the polysaccharide matrix remained on the rock particles until day 100. Furthermore, the microcosm from the contaminated sample MW2 exhibited a higher presence of both microbial cells and polysaccharides on the rock surface (Figure 3h).

The results of assessing the respiratory activity of the microbial cells in both attached and planktonic forms throughout the experiment indicate that biofilm growth in microcosms MW1 and MW2 began within the first day, and the respiratory activity of the planktonic cells was consistently lower than that of the attached cells throughout the experiment (Figure 4a,b).

During the first 15 days, the respiratory activity of microbial cells in the MW2 microcosm from the contaminated sample was consistently higher than that of the MW1 microcosm from the uncontaminated sample. Furthermore, from day 15 to day 40, there was virtually no difference in the respiratory activity between the two microcosms. However, a significant increase in the respiratory activity of the MW2 biofilm was observed on day 40, whereas for the MW1 biofilm, it was seen only on day 70. By day 100, the MW2 biofilm exhibited the highest cell respiration, which had decreased in the MW1 biofilm. These fluctuations can be attributed to changes in microbial diversity after the initial consumption of organic substances and the subsequent transition to anaerobic growth due to oxygen depletion in the vials. Over a period of 30 days, the respiratory activity and visual assessment of biofilm formation correlated. The increase in the respiratory rate on day 40 was not linked to biofilm regrowth, but likely resulted from a shift in the dominant microorganisms due to the depletion of organic substances and the growth of bacteria that consumed the polymer biofilm components. 

The main parameters of biofilm formation by microorganisms in the groundwater samples are presented in Table 1. Comparison of respiratory activity determined with the MTT test revealed that the microbial community from the contaminated site W2 exhibited a higher level of biofilm formation activity compared with the uncontaminated site W1. Additionally, the MW2 biofilm from the contaminated site demonstrated greater stability and remained on the sandy loams for a longer time.

### 3.3. The Monosaccharides’ Composition in the Biofilms

The duration of microbial biofilm development is a crucial factor for bioremediation of underground aquifers because it affects the effectiveness of the process. On day 100 of the experiment, a noticeable decrease in the biofilm area was observed in both sandy loams, although complete disappearance was not recorded.

**Figure 5:**
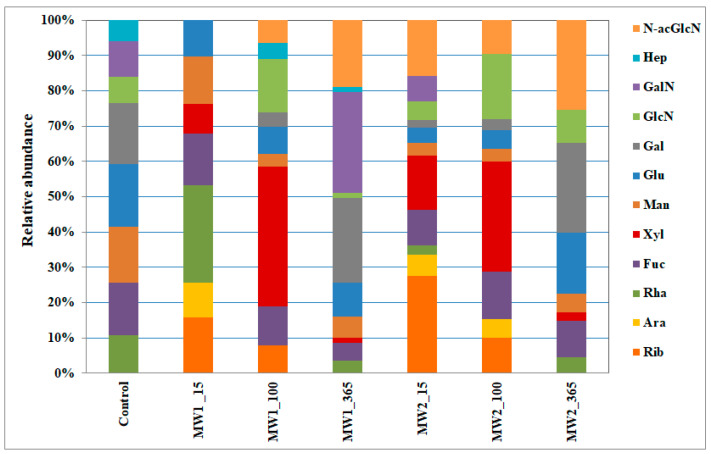
The composition of monosaccharides in the EPS matrix of MW1 and MW2 biofilms, analyzed at days 15, 100, and 365 of the experiment. Designations: N-acGlcN, N-acetylglucosamine; Hep, heptose; GalN, galactoseamine; GlcN, glucosamine; Gal, galactose; Glu, glucose; Man, mannose; Xyl, xylose; Fuc, fucose; Rha, rhamnose; Ara, arabinose; Rib, ribose.

The monosaccharide composition of the MW1 and MW2 biofilms was determined at 15, 100, and 365 days of incubation (Figure 5). A greater diversity of monosaccharides was noted in the MW2 biofilms at day 15 of incubation. A decrease in monosaccharide diversity was observed in both biofilms, MW1 and MW2, at day 100 of cultivation, with a prevalence of xylose and the formation of glucosamine and N-acetylglucosamine. The appearance of glucosamine may be attributed to the death of a large number of cells and the release of cell wall components. The increased overall proportion of xylose in the biofilm matrix composition is associated with its greater resistance to biodegradation compared with other sugars [62]. On day 365, an increase in the proportion of nitrogen-containing monosaccharides in the composition of the biofilms was observed. On day 365, there was an increase in the galactose content in the monosaccharide composition of the MW1 biofilm, in which heptose was present throughout the entire experiment. Heptoses are known as phosphate carriers, which may lead to the formation of additional negatively charged sites for the immobilization of pollutants [63]. 

Based on the obtained results, it can be inferred that the EPS matrix of the biofilms remains intact in the sandy loams even after the cessation of bacterial activity. This finding has the potential to significantly impact the sorption properties of the sandy loams for radionuclides that have an affinity for interacting with organic chelating groups [38,64].

### 3.4. Phylogenetic Diversity of Bacteria in the Biofilms

The formation of biofilms was accompanied by a succession in the microbial community composition. The microbial composition of the MW1 and MW2 biofilms was determined using high-throughput sequencing of the V4 fragment of the 16S rRNA gene at days 7, 12, 40, 70, and 100 of incubation. 

In all analyzed libraries of the 16S rRNA gene fragments, the proportion of Bacteria exceeded 99%, with the proportion of Archaea being consistently low throughout the experiment, ranging from 0.03% to 0.07% in the MW1 biofilms and from 0.07% to 0.23% in the MW2 biofilms. Diversity indices (Table S4) such as CHAO1 and Shannon, which assess the actual (maximum) number of taxa, increased with incubation time for both studied communities. The increased prokaryotic diversity in the biofilms may be a result of changing ecological conditions within the growing biofilms as well as the growth of new populations, probably with different dominant microorganisms. The Simpson dominance index for the MW1 biofilms from W1 reached its maximum value after 7 days, gradually decreasing at the end of the experiment, while for the MW2 biofilms from W2, it reached its peak at 100 days. 

**Figure 6:**
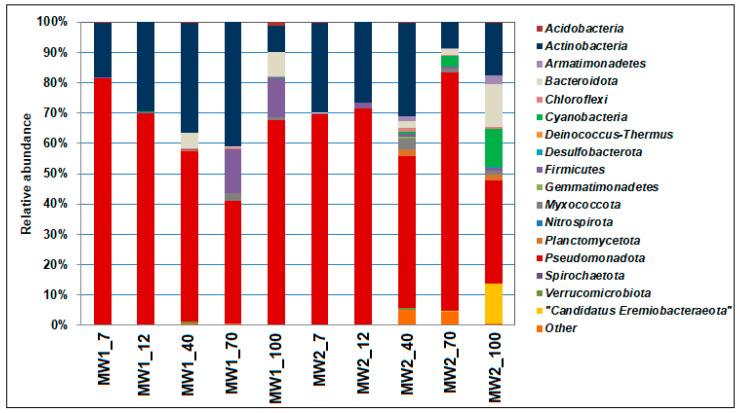
Relative abundance of the fragments of bacterial 16S rRNA gene sequences at the phylum level in the libraries from biofilms obtained from contaminated (W2) and uncontaminated (W1) groundwater samples. The taxa constituting > 1% in each library are listed.

The taxonomic distribution of bacteria at the phylum level is shown in Figure 6. In all analyzed biofilms from samples W1 and W2, the phyla *Pseudomonadota* (34.2–81.3%) and *Actinomycetota* (8.5–41.0%) were predominant. However, the ratio of these dominant phyla changed during cultivation compared with the biofilms formed at 7 days.

With increasing cultivation time, a reverse process occurred: on day 100, the proportion of *Pseudomonadota* increased again (to 67.5%) and the proportion of *Actinomycetota* decreased (to 8.5%). In the MW2 biofilms, from day 7 to day 40, the proportion of *Pseudomonadota* ranged from 69.4% to 50.3%, while *Actinomycetota* ranged from 29.7% to 30.7%. It is worth noting that on day 70, the presence of *Pseudomonadota* increased (to 78.6%) and that of *Actinomycetota* decreased (to 8.6%), and by day 100, a reverse process had occurred, with a significant increase in the presence of bacteria from the phyla *Bacteroidota*, “*Cyanobacteria*”, and unclassified *Candidatus* Eremiobacteraeota. The proportion of bacteria of the phylum *Bacillota* increased in the MW1 biofilms, reaching a maximum (14.3%) on day 70 of cultivation, while in the MW2 biofilms, it varied slightly (from 0.3% to 1.6%). The investigated communities also differed in the content of bacteria from the phylum “*Cyanobacteria*”, which made up a significant proportion in the MW2 biofilms (0.8% to 12.3% after 40 days of cultivation) but were absent in the MW1 biofilms.

**Figure 7:**
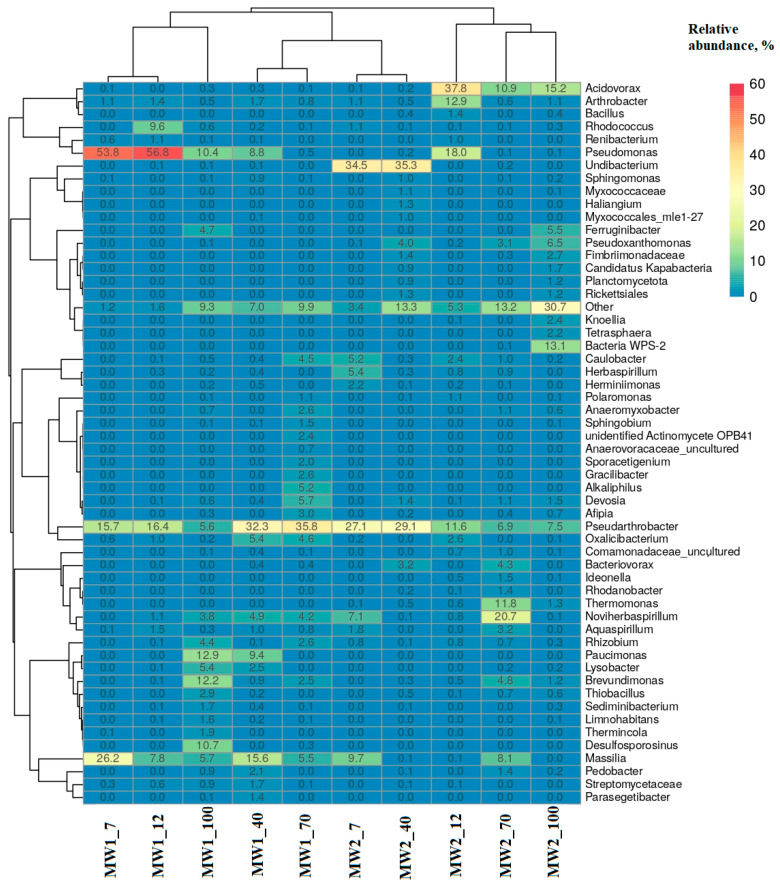
The heatmap of the most prevalent genera in the libraries of 16S rRNA gene fragments from prokaryotic MW1 and MW2 biofilms. The numbers on the diagram represent the percentages of sequences from each sample in relation to the total number of sequences in the library. Columns are clustered using correlation distance and average linkage. The double hierarchal tree shows the distribution of microorganisms in these samples.

The taxonomic composition of the bacterial genera most commonly found in biofilms is presented on a heatmap generated using the ClustVis program (Figure 7). In the MW1 biofilms, the genus *Pseudomonas* dominated during the first 12 days of cultivation, but by day 40, it was replaced by the dominant representatives of the actinobacterial genus *Pseudarthrobacter*. By day 100 of the experiment, the dominant genera in the biofilms included *Paucimonas* (12.9%), *Brevundimonas* (12.2%), *Desulfosporosinus* (10.7%), and *Pseudomonas* (10.4%). In the MW2 biofilms, the proportion of *Pseudomonas* also reached its peak (18%) on day 12, but thereafter, its proportion did not exceed 0.2%. However, the dominant bacteria were *Undibacterium* on days 7 and 40 of incubation, *Acidovorax* on days 12 and 100, and *Noviherbaspirillum* on day 70. Throughout the entire experiment, *Pseudarthrobacter* was responsible for a significant proportion in both the MW1 and MW2 biofilms. The emergence of sulfate-reducing *Desulfosporosinus* bacteria in the MW1 biofilms, which are often found in soil, is worth noting. Representatives of this genus are resistant to high concentrations of metals such as Ni(II), Co(II), and Cu(II) [65,66,67].

The genus *Noviherbaspirillum* includes bacteria capable of autotrophic growth and nitrate reduction [68]; nitrate was present in the liquid radioactive waste. Other bacteria in the investigated microbial communities were also capable of dissimilatory nitrate reduction and predominated in the biofilms from the contaminated groundwater samples (such as *Acidovorax*, *Pseudomonas*, etc.).

Thus, it is evident that over time, changes in the composition of microorganisms occurred within the examined biofilms.

Microbial interaction involving exometabolite production is an integral part of biofilm formation. A well-studied mechanism of microbial interaction is the quorum sensing (QS) system, which consists of a response-stimulating system dependent on cell concentration. The changes in QS profiles in the MW1 biofilms may indicate that QS is necessary for biofilm formation at the initial stage, while on day 70, there may have been a breakdown of the polysaccharide matrix, accompanied by biofilm lysis and restructuring. The QS indicator remained more stable for the MW2 biofilms, suggesting a more resilient biofilm formed by microorganisms in the contaminated sample, where components function as a coordinated, organized, and unified community (Table S5). 

Using KEGG analysis, we predicted that the functional characteristics of the microorganisms in the MW1 and MW2 biofilms were similar and remained nearly unchanged during biofilm cultivation (Figure S1). Bacterial components of the biofilms potentially contribute significantly to the metabolism of benzoate, a central metabolite in the degradation of aromatic compounds. Enzymes responsible for QS were highly represented in the bacterial components of the biofilms. 

Table S6 provides a list of the key bacteria involved in nitrogen metabolism. In the early stages of the MW1 biofilm formation, bacteria of the genus *Pseudomonas* presumably made the greatest contribution. By the 40th day, members of the genus *Noviherbaspirillum* joined them. In the MW2 biofilms, the potential contribution of *Pseudomonas* to nitrogen compound transformations was considerably lower, with *Acidovorax*, *Noviherbaspirillum*, and *Pseudoxanthomonas* presumably making the major contribution. The high nitrate concentration and lower pH values in the contaminated W2 sample were probably unfavorable for the growth of *Pseudomonas* bacteria. 

A visual representation of the differences in the composition of the MW1 and MW2 biofilms over time is provided using the principal component analysis method (Figure S2), based on the relative abundance of operational taxonomic units of prokaryotic 16S rRNA genes, as well as by comparing OTUs in the libraries using Venn diagrams (Figure S3).

### 3.5. Pure Cultures of Bacteria Isolated from Biofilms

From the obtained biofilms, 12 strains of bacteria were isolated in pure cultures. The similarity of the 16S rRNA gene sequences of the isolates to the closest genes in GenBank exceeded 99%, allowing the strains to be classified as members of known species: *Bacillus proteolyticus* (strains I-16-d, I-25-h, II-15-k, I-15-n, and I-15-o), *Paenibacillus glucanolyticus* (strains II-2181-a, II-2181-b, II-2182-c, and II-25-e), and *Microbacterium flavescens* (strains II-25-f, II-217-g, and II-25-m). The isolated strains were Gram-positive aerobic or facultatively anaerobic rods. Bacteria *P. glucanolyticus* [69,70] and *M. flavescens* [71] are known to be capable of nitrate reduction. Respiratory activity and the amount of organic matter produced during growth in liquid media (planktonic culture) and as biofilms on sandy loams for 7 days were determined for *P. glucanolyticus,* II-2181-a and II-25-e strains; *B. proteolyticus,* I-16-d; and *M. flavescens,* II-25-m (Figure S4). 

The respiratory activity of the biofilms of all four strains was shown to be significantly higher than that of the planktonic cultures. The biofilms of *M. flavescens* strain II-25-m exhibited the highest respiratory activity, while the highest production of organic matter was observed for *B. proteolyticus* strain I-16-d. A visual assessment of the biofilm formed on sandy loam particles by the studied bacteria revealed differences in the amount of polysaccharide matrix produced. The strains *P. glucanolyticus* II-2181-a and II-25-e differed from each other in the analyzed parameters. The respiratory activity of the planktonic and biofilm cultures, as well as the amount of organic matter produced during bacterial growth as a biofilm, were higher in strain II-25-e compared with strain II-2181-a by 1.5 and 1.2 times, respectively.

### 3.6. Reduction of Nitrate Ions and Heavy Metals by the Biofilm Microbial Community and Pure Bacterial Cultures

**Figure 8:**
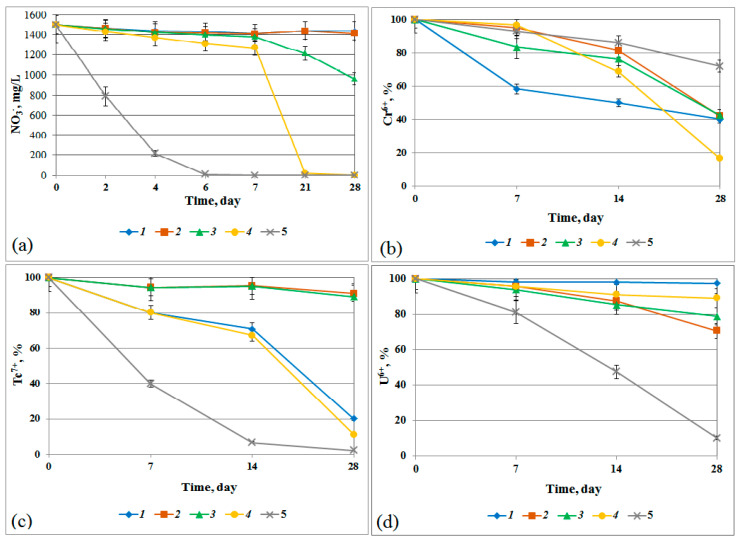
The reduction of nitrate (**a**), chromate (**b**), pertechnetate ions (**c**), and uranyl (**d**) by the pure bacterial cultures *P. glucanolyticus* II-2181-a (1), *P. glucanolyticus* II-25-e (2), *M. flavescens* II-25-m (3), and *B. proteolyticus* I-16-d (4), and by the MW2 biofilms (5).

Figure 8 shows the dynamics of oxidant reduction (NO_3_^−^ to N_2_, UO_2_^2−^ to UO_2_, CrO_3_ to Cr(OH)_3_, and TcO_4_ to TcO_2_) by the microbial community of the MW2 biofilm and by pure cultures of *P. glucanolyticus* strains II-2181-a and II-25-e, *B. proteolyticus* I-16-d, and *M. flavescens* II-25-m.

The highest reduction rate was observed for nitrate ions by MW2 biofilms. The pure cultures reduced nitrate, uranyl, and pertechnetate ions significantly slower than the microbial community of the MW2 biofilm. This is associated with the multispecies composition of the biofilm, the phenomenon of quorum sensing, as well as possible protective functions of the exopolysaccharide matrix of the biofilm under toxic stress [72].

The strain *B. proteolyticus* I-16-d reduced all nitrate ions present in the medium within 21 days. This strain also reduced more Cr^6+^ ions in the medium than the other tested strains. Unlike the MW2 biofilm, the individual strains showed weak reduction of uranyl ions. Effective reduction of pertechnetate ions was observed not only with the MW2 biofilms but also with the strains *B. proteolyticus* I-16-d and *P. glucanolyticus* II-2181-a, which reduced over 80% of the present Tc^7+^ ions in the medium, while the other two strains showed weak activity toward Tc^7+^ ions.

The strain *B. proteolyticus* I-16-d, which is capable of forming biofilms and reducing nitrate, technetium, and chromium, as well as the strains *P. glucanolyticus* II-25-e, which was able to reduce uranium, and *P. glucanolyticus* II-2181-a, which reduced technetium and chromium, may be used as components in permeable barriers for in situ bioremediation of contaminated groundwater. The *M. flavescens* II-25-m strain is also noteworthy due to its increased metabolic activity in biofilm formation and resistance to nitrate, uranium, and technetium, although it reduces them weakly. 

## 4. Discussion

The long-term effects of groundwater pollution with liquid radioactive waste components have an impact on the taxonomic diversity and physiological activity of the indigenous microbial communities, including their ability to form biofilms on mineral rocks. Biofilms are the predominant form of existence for many bacteria. As a rule, a multispecies microbial community is found in biofilms. Using next-generation sequencing (NGS) technologies has demonstrated that only up to 5% of bacteria have been cultured by the current cultural methods [73]. The NGS sequencing method makes it possible to trace changes in the composition of biofilm microbial communities. The species composition, structure, and morphology of biofilms can vary depending on the growth rate of the microorganisms and the availability of nutrients [74].

The method of in situ bioremediation of groundwater with complex contamination involves stimulating the indigenous microflora with inexpensive organic substrates to establish conditions for the removal or immobilization of pollutants. A key step is the microbial consumption of oxidants (oxygen, nitrate), which allows for the development of reducing conditions facilitating the formation of reduced, less soluble phases of actinides and technetium and their subsequent immobilization within biogenic iron sulfide and carbonate authigenic mineral phases. This approach has proven effective in the remediation of groundwater contaminated with nitrate and actinides in the vicinity of radiochemical enterprises in the United States and China [16,75,76,77]. Recent studies [19,24,78] demonstrate the high technological potential of this approach.

In a field experiment conducted near the preserved surface basin for liquid radioactive waste at the Siberian Chemical Combine (Tomsk region, Russia), an organic substrate (milk whey) was added to stimulate the in situ growth of the microbial community in the upper aquifer horizon [23]. The activation of bacterial metabolic activity resulted in the temporary removal from the groundwater horizon of nitrate ions, which are among the major components of liquid radioactive waste, with the concentrations exceeding permissible levels in the burial zone. However, after 12 months, the nitrate ion concentration returned to its previous levels. Because the formation of a stable biogeochemical barrier during in situ bioremediation largely depends on the formation of microbial biofilms on the rocks of the aquifer horizons, this study focused on laboratory modeling of biofilm formation on rocks sampled from areas with different anthropogenic loads in the vicinity of the field experiments at the Siberian Chemical Combine. Differences in biofilm formation on sandy loam rocks by microorganisms from groundwater wells within and outside the contamination zone were observed. 

Through a prolonged experiment involving a single acetate stimulation of the microbial community, it was observed that the maximum biofilm area on sandy loams and the highest respiratory activity of the biofilms were achieved by day 15 of cultivation. Subsequently, there was a gradual decrease in the exopolysaccharide matrix of the biofilm, which may be attributed to the rapid consumption of easily accessible organic substrates by the microorganisms. For the biofilms from the contaminated sample, a regrowth in respiratory activity was observed on day 40 of the experiment, while for the uncontaminated sample, this regrowth occurred on day 70. Analysis of the monosaccharide composition of the exopolysaccharide matrix revealed that the highest diversity of monosaccharides in the biofilm (including ribose, arabinose, rhamnose, fucose, xylose, mannose, glucose, galactose, glucosamine, galactosamine, and N-acetylglucosamine) was also observed on day 15 of the experiment. Over time, there was a decrease in the content of simple carbohydrates and an increase in the contribution of xylose, a biodegradation-resistant carbohydrate.

In our experiment, the stimulation of the microbial community with a simple substrate (acetate) led to the development of a biofilm dominated by organotrophic bacteria. These bacteria were capable of utilizing the EPS matrix and other components of the biofilms after depletion of the soluble substrate. Changes in the dominant genera composition in the biofilms during cultivation were confirmed by molecular biological studies in which libraries of V4 fragment sequences of the 16S rRNA gene of prokaryotes were obtained. The taxonomic composition of the prokaryotes in the biofilms obtained from wells with varying degrees of contamination showed significant differences. For instance, the MW2 biofilm sample from the contaminated zone W2 exhibited greater bacterial diversity and a higher number of dominant bacterial genera, especially in the later stages of biofilm cultivation, when the proportion of representatives of the phyla *Bacteroidota*, “*Cyanobacteria*”, and *Candidatus* Eremiobacteraeota increased. However, *Pseudomonadota* (34.2–81.3%) and *Actinomycetota* (8.5–41.0%) were dominant in all analyzed biofilms. Nevertheless, unlike the MW1 biofilms, the MW2 biofilms had a slightly lower representation of the phylum *Bacillota* and included representatives of the phylum “*Cyanobacteria*”. The QS indicator remained more stable for the bacteria in the MW2 biofilms compared with the MW1 biofilms, indicating formation of more resilient biofilms by the microbial community under contaminated conditions, despite its smaller surface coverage. To create a long-lasting barrier for in situ remediation in areas of technogenic impact, it is necessary to support the development of resilient biofilms by injecting organic compounds into the underground horizons.

The protective role of biofilms in establishing a barrier in an area of radioactive waste contamination is determined by the ability of their components to reduce nitrates. The results of determining the composition of the biofilm microbial communities using metagenomic analysis based on the analysis of 16S rRNA gene fragments showed the presence of bacteria capable of denitrification as well as of the nitrate metabolism pathways in the functional profile of the community. 

It is important to note that microorganisms capable of reducing uranyl, chromate, and pertechnetate ions to less mobile forms were found in the composition of the biofilms. It was shown that nitrate, uranyl, and pertechnetate ions were reduced faster by the biofilm microorganisms than by pure cultures of *P. glucanolyticus* II-2181-a, *P. glucanolyticus* II-25-e, *M. flavescens* II-25-m, and *B. proteolyticus* I-16-d; this can be explained by the wide species diversity of the biofilm, the phenomenon of quorum sensing, and the protective function of the exopolysaccharide matrix of the biofilm during toxic stress. The data obtained are summarized in Table S7.

## 5. Conclusions

In the present study, the microbial diversity in the biofilms formed on sandy loams, sampled from the water-bearing horizon in the clean area and in the area contaminated with nitrate and radionuclides near a surface repository of liquid radioactive waste (Russia), was assessed with 16S rRNA gene sequencing. The indigenous, metabolically heterogeneous microbial community of the groundwater was capable of forming stable multispecies biofilms on mineral substrates as well as of removing nitrate ions from the environment by reducing them to molecular nitrogen, thus establishing the conditions for reduction of uranyl, chromate, and pertechnetate ions to less soluble forms, thereby decreasing their migration with groundwater.

The impact of pollution on the taxonomic composition of microorganisms is evident in the monosaccharide content of EPS, the prolonged development of biofilms, the increased activity of microorganisms in the restored biofilm on sandy loam surfaces, and the rate of pollutant removal. Similar characteristics in the biofilm development cycle of both clean and contaminated areas, the presence of *Pseudomonas* spp. bacteria capable of restoring the dissolved components of liquid radioactive waste, and the prevalence of resistant monosaccharides in the EPS composition indicate that the biofilms in these communities will play a crucial role as a biogeochemical barrier in underground aquifers, preventing the spread of pollution. The results of the study have both scientific and practical significance, as they demonstrate that activating the biofilm growth of the microbial community to establish biogeochemical barriers can lead to decreased nitrate ion content in contaminated groundwater and to an increase in radionuclide immobilization by hindering their mobility.

## Figures and Tables

**Table 1:** The growth parameters of microbial biofilms on sandy loams in a laboratory experiment.

Sample	Parameters	Incubation Time, Days			
		1	20	40	100
MW1	MTT test	0.4	0.8	1.2	1.5
	Proteins, mg/L	0.8	6.4	4.2	7.3
	C_org_, mass. %	1.6	9.0	2.4	0.2
	Polysaccharides, %	0.3	19.8	7.8	2.8
	Nucleic acids, %	0.2	4.2	2.7	1.8
MW2	MTT test	0.8	0.9	2.1	2.5
	Proteins, mg/L	0.9	2.8	5.6	9.9
	C_org_, mass. %	1.2	8.5	1.2	0.4
	Polysaccharides, %	0.3	23.6	10.9	3.3
	Nucleic acids, %	0.5	3.8	11.2	3.4

## Data Availability

The 16S rRNA gene sequences of pure cultures have been deposited to GenBank under accession Nos. MK567698, MK567783, and MK571249. The 16S rRNA gene fragment sequences of bacteria from the biofilms are available in the NCBI SRA database under the following accession number: SUB14021502, BioProject PRJNA1054355.

## Supplementary Materials

The following supporting information can be downloaded at: https://www.mdpi.com/article/10.3390/microorganisms12020275/s1, Figure S1: Heatmap showing the predicted functional profiles of the studied microbial communities based on the KEGG database; Figure S2: Comparison of the composition of microbial communities in the MW1 and MW2 microcosms using the principal component analysis method based on the relative number of operational taxonomic units of 16S rRNA genes; Figure S3: Venn diagram showing the logical relation between OTUs of 16S rRNA genes of the bacterial community in microcosms MW1 (a) and MW2 (b); Figure S4: Respiratory activity (MTT test) of planktonic cells and biofilms of the bacteria *Paenibacillus glucanolyticus* II-2181-a, *Paenibacillus glucanolyticus* II-25-e, *Bacillus proteolyticus* I-16-d, and *Microbacterium flavescens* II-25-f and the proportion of organic matter on the ground (%); Table S1: Chemical composition of water taken from observation wells W1 and W2; Table S2: The content of radionuclides in groundwater samples; Table S3: Sandy rock composition, % mass; Table S4: Diversity indices in the 16S rRNA gene libraries of bacterial communities of biofilms in microcosms MW1 and MW2; Table S5: Key microorganisms involved in QS in microcosms MW1 and MW2, %; Table S6: Key microorganisms involved in nitrogen metabolism in microcosms MW1 and MW2, %. Table S7: Comparison and summary of the data obtained.
