# Supplementary material for: Microbial and Monosaccharide Composition of Biofilms Developing on Sandy Loams from an Aquifer Contaminated with Liquid Radioactive Waste
Source: Microorganisms. 2024 Jan 28;12(2):275. doi: 10.3390/microorganisms12020275 (PMC10892373; doi:10.3390/microorganisms12020275)
Supplement: Supplementary file 1 [file microorganisms-12-00275-s001.zip › microorganisms-2827004-supplementary.pdf]

microorganisms

Supplementary Materials

Microbial and Monosaccharide Composition of Biofilms Developing on Sandy Loams from an Aquifer Contaminated with Liquid Radioactive Waste

Tamara L. Babich [1], Nadezhda M. Popova [2], Diyana S. Sokolova [1], Andrei V. Perepelov [3], Alexey V. Safonov [2] and Tamara N. Nazina [1,*]

[1] Winogradsky Institute of Microbiology, Research Center of Biotechnology, Russian Academy of Sciences, Moscow, 119071, Russia; microb101@yandex.ru (T.L.B.); sokolovadiyana@gmail.com (D.S.S.)
[2] A.N. Frumkin Institute of Physical Chemistry and Electrochemistry, Russian Academy of Sciences, 119071 Moscow, Russia; nm.popova.ipce.ras@gmail.com (N.M.P.); alexeysafonof@gmail.com (A.V.S.)
[3] N.D. Zelinsky Institute of Organic Chemistry, Russian Academy of Sciences, 119991 Moscow, Russia; andreivperepelov@gmail.com
* Correspondence: nazina@inmi.ru, Tel.: +7-499-135-0341

This file includes:

Figures S1 to S4

Tables S1 to S7

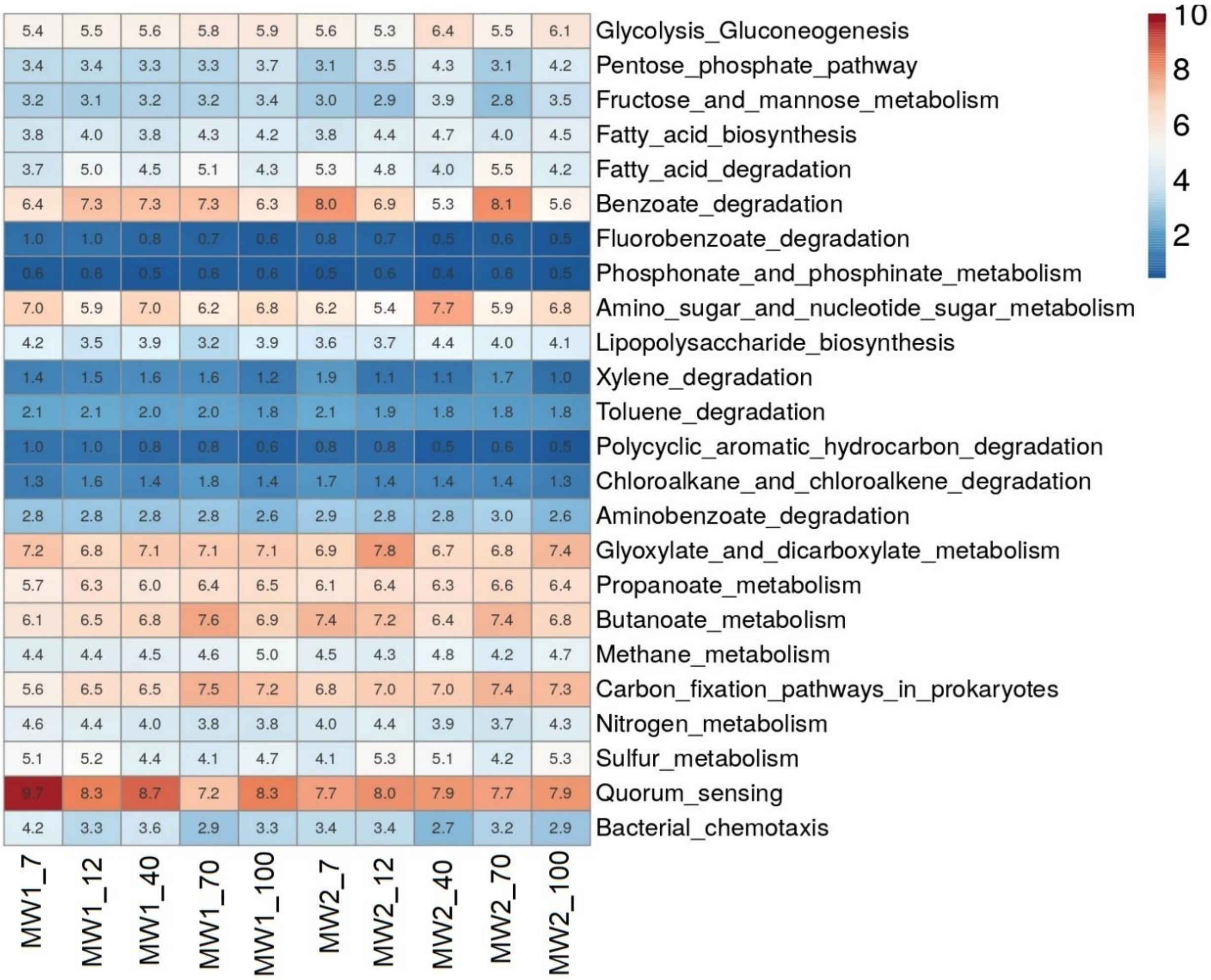

Figure S1. The heatmap showing the predicted functional profiles of studied microbial communities based on the KEGG Database.

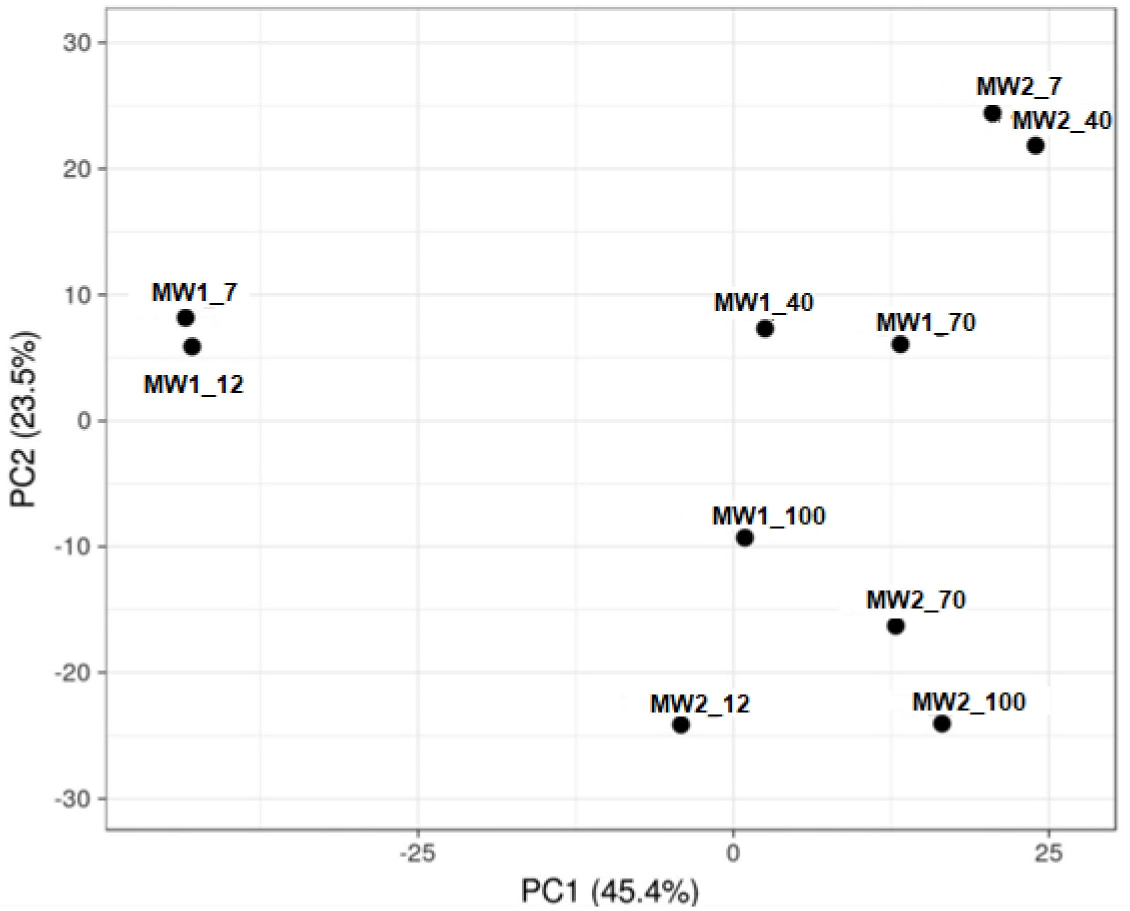

Figure S2. Comparison of the composition of microbial communities in the MW1 and MW2 microcosms using the principal component analysis method based on the relative number of operational taxonomic units of 16S rRNA genes.

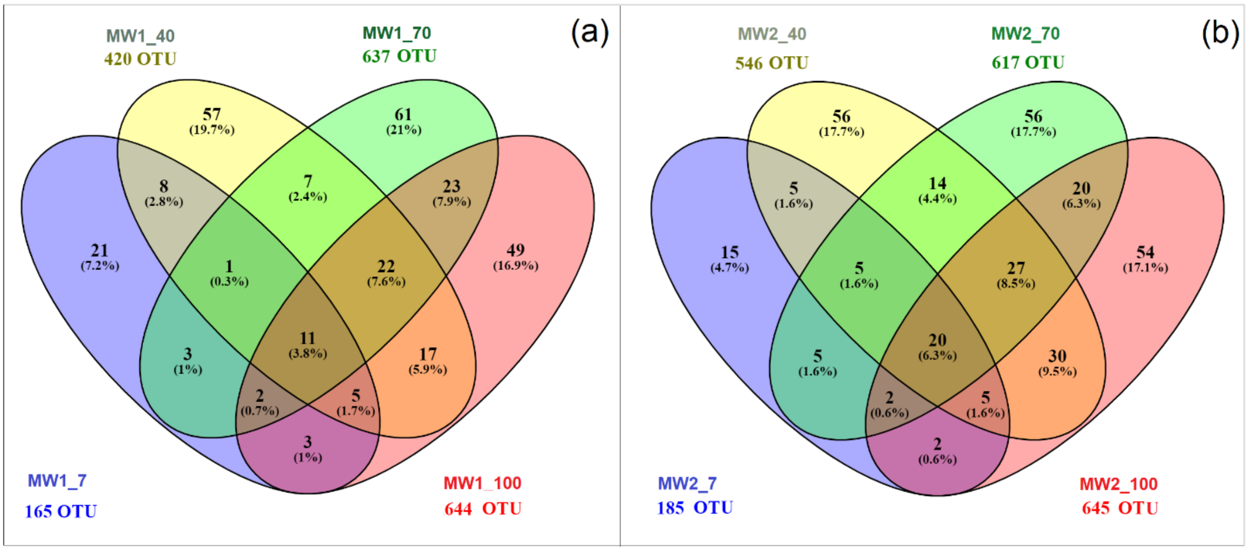

Figure S3. Venn diagram, showing the logical relation between OTUs of 16S rRNA genes of the bacterial community in microcosms MW1 (a) and MW2 (b).

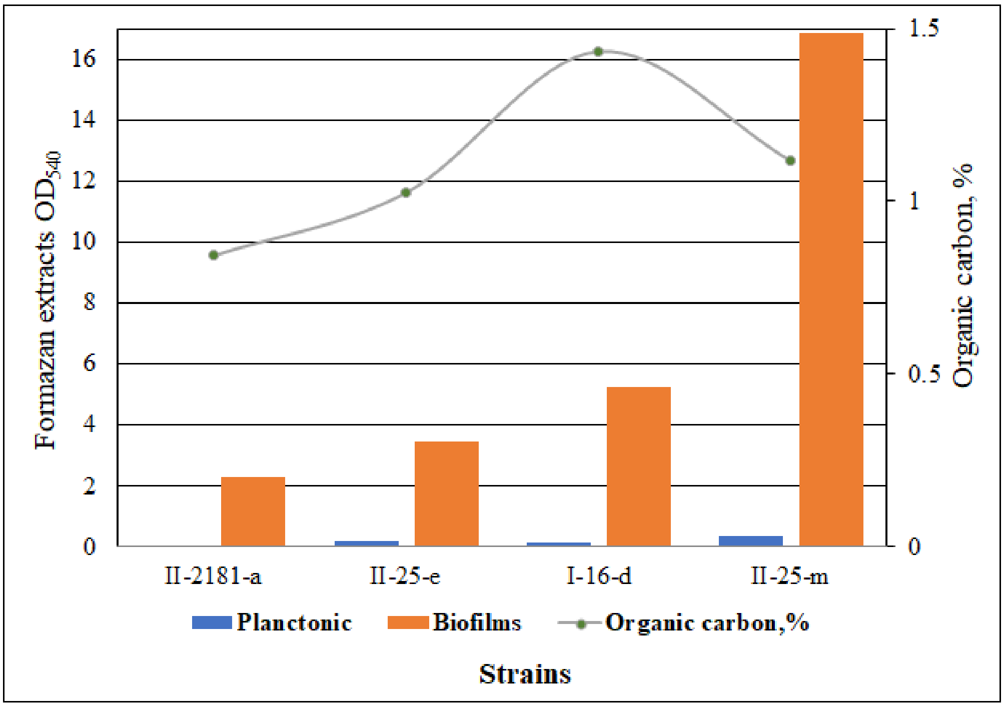

Figure S4. Respiratory activity (MTT test) of planktonic cells and biofilms of bacteria *Paenibacillus glucanolyticus* II-2181-a, *Paenibacillus glucanolyticus* II-25-e, *Bacillus proteolyticus* I-16-d, and *Microbacterium flavescens* II-25-f and the proportion of organic matter on the ground (%).

Table S1. Chemical composition of water taken from observation wells W1 and W2.

Well No.	Total salinity, mg/L	pH	Content, mg/L								
			Fe	Na^+	Ca^{2+}	Mg^{2+}	NH_4^+	NO_3^-	SO_4^{2-}	HCO_3^-	CO_2
W1	109	6.4	2.38	3.41	15.4	2.8	<0,5	0.77	0.84	67.1	19.4
W2	5070	6.2	<0.02	760	375	63.4	9.25	3280	46	140.3	64

Table S2. The content of radionuclides in groundwater samples.

Well No.	Radioactivity, Bq/L					
	^{137}Cs	^{90}Sr	^{99}Tc	$\Sigma\beta$	$\Sigma\alpha$	U(sum)
**W1**	0.05	0.04	<0.1	<1.9	0.039	<0.01
**W2**	0.11	345.7	16.5	322	0.051	9.18

Table S3. Sandy rock composition, % mass.

Rock sample	Na_2O	MgO	Al_2O_3	SiO_2	K_2O	CaO	TiO_2	MnO	Fe_2O_3	P_2O_5	S
W1	1.68	1.14	8.64	79.32	1.69	1.46	0.39	0.038	2.75	0.07	0.09
W2	3.18	2.80	7.78	74.20	1.98	4.45	0.01	0.001	2.15	0.05	2.78

Table S4. Diversity indices in the 16S rRNA gene libraries of bacterial communities of biofilms in microcosms MW1 and MW2.

Parameter	Libraries of 16S rRNA gene fragments									
	MW1					MW2				
	Incubation time, day					Incubation time, day				
	7	12	40	70	100	7	12	40	70	100
Number of sequences (reads)	6472	7992	5441	6108	6107	5206	4855	6556	5456	5771
Number of OTUs	153	298	420	637	644	185	319	546	617	153
CHAO1	360.8	620.0	1028.2	1549.7	1374.7	422.4	761.2	1605.1	1536.2	1584.8
Shannon–Weaver diversity index (*H*)	1.79	2.56	3.24	3.54	4.15	2.34	2.61	2.88	4.14	4.04
Simpson's index	0.30	0.22	0.14	0.15	0.05	0.21	0.21	0.21	0.05	0.30
Good's coverage, (%)	99	98	95	93	94	98	96	95	93	93

Table S5. Key microorganisms involved in QS in microcosms MW1 and MW2, %.

Bacteria, genus	MW1_7	MW1_12	MW1_40	MW1_70	MW1_100	MW2_7	MW2_12	MW2_40	MW2_70	MW2_100
Massilia	67.9	76.5	24.8	2.3	23.5	0.1	31.8	2.0	0.1	0.4
Acidovorax	30.6	9.7	40.8	22.5	11.9	36.7	0.2	1.5	14.3	0.1
Noviherbaspirillum	0.0	0.0	0.5	0.2	0.6	0.1	48.6	1.9	15.1	45.7
Brevundimonas	0.0	1.3	12.0	16.3	7.5	25.2	1.2	0.7	34.2	0.4
Arthrobacter	0.0	0.1	1.3	5.7	14.3	0.0	0.5	2.0	4.8	2.7
Rhodococcus	0.6	0.8	2.0	1.6	0.5	1.9	9.8	2.6	0.5	2.0
Pseudoxanthomonas	0.0	8.4	0.4	0.3	0.9	3.1	0.1	1.1	0.1	0.8
Thermomonas	0.0	0.0	0.0	0.0	0.1	0.1	0.2	25.0	3.3	14.9
Herbaspirillum	0.0	0.0	0.0	0.0	0.0	0.2	0.5	2.4	10.5	2.5
Rhizobium	0.0	0.3	0.9	0.1	0.5	17.8	1.1	3.1	1.3	0.0
Desulfosporosinus	0.0	0.1	0.2	6.7	5.8	1.9	0.8	0.7	0.8	0.7
Lysobacter	0.0	0.0	0.0	0.7	12.6	0.0	0.0	0.0	0.0	0.0
Aquaspirillum	0.0	0.1	4.5	0.1	8.0	0.1	0.0	0.2	0.3	0.6
Anaeromyxobacter	0.1	1.3	1.7	2.1	0.4	4.6	0.0	0.0	3.8	0.0
Afipia	0.0	0.0	0.0	8.5	1.1	0.0	0.0	0.2	1.5	1.8
Thiobacillus	0.0	0.0	0.1	9.3	0.4	0.1	0.0	1.4	0.5	2.1
Streptomycetaceae	0.0	0.0	0.3	0.1	4.0	0.1	0.1	3.7	0.8	1.6
Chitinophagaceae	0.3	0.7	3.7	0.3	1.7	0.0	0.1	0.7	0.0	0.0
Alkaliphilus	0.0	0.0	0.0	0.0	0.2	0.0	0.0	1.7	0.2	7.9
Fimbriimonadaceae	0.0	0.0	0.0	9.3	0.0	0.0	0.0	0.0	0.0	0.0
Herminiimonas	0.0	0.0	0.0	0.0	0.0	0.0	0.0	7.6	0.3	5.1
Pedobacter	0.0	0.0	0.8	0.1	0.3	5.2	0.2	0.8	0.1	0.0
Caulobacter	0.0	0.0	2.2	0.0	0.7	0.0	0.0	0.4	1.0	0.3
Polaromonas	0.0	0.0	0.1	2.4	0.1	2.5	0.5	0.4	0.2	0.1

Table S5. Cont.

Bacteria, genus	MW1_7	MW1_12	MW1_40	MW1_70	MW1_100	MW2_7	MW2_12	MW2_40	MW2_70	MW2_100
Comamonadaceae	0.0	0.0	0.0	3.2	0.2	0.0	1.3	0.5	0.0	0.2
Rhodanobacter	0.0	0.0	0.8	0.2	0.1	0.0	0.8	0.2	1.1	0.2
Bacteriovorax	0.0	0.0	0.0	0.0	0.0	0.0	0.1	1.4	2.1	0.1
Sphingomonas	0.0	0.0	0.2	0.3	0.0	0.0	0.0	5.4	1.2	0.0
Renibacterium	0.0	0.0	1.3	0.2	0.1	0.1	0.0	5.8	0.1	0.4
Limnohabitans	0.0	0.0	0.0	0.0	0.0	0.0	0.5	0.0	1.6	0.1
Sphingobium	0.0	0.0	0.3	0.3	1.8	0.0	0.0	0.2	0.0	0.2
Knoellia	0.0	0.0	0.2	3.7	0.1	0.0	0.0	0.1	0.0	0.1
Myxococcaceae	0.0	0.0	0.0	0.0	0.0	0.1	0.0	9.9	0.0	0.0
Tetrasphaera	0.0	0.0	0.1	0.0	0.0	0.0	0.0	8.5	0.0	0.4
Thermincola	0.0	0.0	0.3	0.1	1.1	0.0	0.0	0.3	0.0	0.3
Bacillus	0.0	0.0	0.0	0.0	1.5	0.0	0.0	0.0	0.0	0.0
Devosia	0.0	0.0	0.0	0.0	0.0	0.0	0.7	1.2	0.0	0.4
Bdellovibrionaceae	0.0	0.0	0.1	1.3	0.1	0.0	0.0	0.8	0.1	0.3
Sporacetigenium	0.0	0.0	0.0	0.0	0.0	0.0	0.0	5.6	0.0	0.0

Table S6. Key microorganisms involved in nitrogen metabolism in microcosms MW1 and MW2, %.

**Bacteria, genus**	**MW1_7**	**MW1_12**	**MW1_40**	**MW1_70**	**MW1_100**	**MW2_7**	**MW2_12**	**MW2_40**	**MW2_70**	**MW2_100**
Pseudomonas	72.4	75.2	27.9	2.3	24.8	0.1	29.8	2.1	0.1	0.4
Massilia	25.8	7.5	36.2	17.5	9.9	29.8	0.1	1.2	12.2	0.1
Acidovorax	0.0	0.0	0.6	0.2	0.6	0.2	46.4	2.0	16.7	44.5
Noviherbaspirillum	0.0	1.2	12.9	15.4	7.6	24.9	1.1	0.7	35.8	0.3
Arthrobacter	0.9	1.2	3.4	2.3	0.7	2.9	13.6	4.0	0.8	2.8
Rhodococcus	0.0	12.0	0.6	0.4	1.4	4.6	0.2	1.7	0.1	1.1
Rhizobium	0.0	0.1	0.4	9.7	9.0	2.8	1.1	1.0	1.3	0.9
Brevundimonas	0.0	0.1	0.9	3.4	9.1	0.0	0.3	1.3	3.1	1.5
Desulfosporosinus	0.0	0.0	0.0	0.8	16.4	0.0	0.0	0.0	0.0	0.0
Pseudoxanthomonas	0.0	0.0	0.0	0.0	0.1	0.1	0.2	22.2	3.1	12.1
Herbaspirillum	0.0	0.2	0.9	0.1	0.4	15.9	0.9	2.8	1.3	0.0
Thermomonas	0.0	0.0	0.0	0.0	0.0	0.2	0.4	2.0	9.1	1.9
Caulobacter	0.0	0.0	0.4	6.9	0.4	7.7	1.4	1.2	0.7	0.3
Aquaspirillum	0.1	1.0	1.6	1.7	0.3	3.9	0.0	0.0	3.4	0.0
Lysobacter	0.0	0.0	2.9	0.0	4.8	0.0	0.0	0.1	0.2	0.3
Anaeromyxobacter	0.0	0.0	0.0	7.1	1.0	0.0	0.0	0.1	1.4	1.5
Afipia	0.0	0.0	0.1	8.8	0.4	0.1	0.0	1.4	0.6	1.9
Thiobacillus	0.0	0.0	0.4	0.1	4.3	0.1	0.1	4.0	0.9	1.6
Chitinophagaceae	0.0	0.0	0.0	0.0	0.3	0.0	0.0	2.2	0.3	9.3
Streptomycetaceae	0.3	0.5	3.5	0.3	1.5	0.0	0.1	0.6	0.0	0.0
Alkaliphilus	0.0	0.0	0.0	10.7	0.0	0.0	0.0	0.0	0.0	0.0
Herminiimonas	0.0	0.0	1.1	0.1	0.3	6.4	0.2	1.0	0.1	0.0
Fimbriimonadaceae	0.0	0.0	0.0	0.0	0.0	0.0	0.0	8.3	0.3	5.2
Polaromonas	0.0	0.0	0.0	3.4	0.3	0.0	1.3	0.5	0.0	0.2

Table S6. Cont.

Bacteria, genus	MW1_7	MW1_12	MW1_40	MW1_70	MW1_100	MW2_7	MW2_12	MW2_40	MW2_70	MW2_100
Pedobacter	0.0	0.0	2.2	0.0	0.7	0.0	0.0	0.4	0.9	0.3
Comamonadaceae	0.0	0.0	0.9	0.2	0.1	0.0	0.8	0.2	1.3	0.2
Bacteriovorax	0.0	0.0	0.3	0.3	0.0	0.0	0.0	7.0	1.7	0.0
Rhodanobacter	0.0	0.0	0.0	0.0	0.0	0.0	0.1	1.3	2.1	0.1
Sphingomonas	0.0	0.0	1.4	0.2	0.1	0.1	0.0	5.6	0.1	0.4
Limnohabitans	0.0	0.0	0.3	0.3	1.9	0.0	0.0	0.2	0.0	0.2
Haliangium	0.0	0.0	0.0	0.0	0.0	0.1	0.0	10.3	0.0	0.0
Sediminibacterium	0.0	0.0	0.4	0.1	1.4	0.0	0.0	0.4	0.0	0.4
Sphingobium	0.0	0.0	0.2	3.1	0.1	0.0	0.0	0.1	0.0	0.1
Thermincola	0.0	0.0	0.0	0.0	1.8	0.0	0.0	0.0	0.0	0.0
Sporacetigenium	0.0	0.0	0.0	3.2	0.0	0.0	0.0	0.0	0.0	0.0
Myxococcaceae	0.0	0.0	0.0	0.0	0.0	0.0	0.0	6.3	0.0	0.3
Bacillus	0.0	0.0	0.0	0.0	0.0	0.0	0.6	1.1	0.0	0.3
Devosia	0.0	0.0	0.1	1.2	0.1	0.0	0.0	0.8	0.1	0.3
Bdellovibrionaceae	0.0	0.0	0.0	0.0	0.0	0.0	0.0	5.5	0.0	0.0

Table S7. Comparison and summary of the data obtained.

Stage	MW1				MW2			
	Biofilm / planctonic cells activity	Biofilm cover-age, %	Main mono-saccha-rides	Dominant taxa	Biofilm / planctonic cells activity	Biofilm cover-age, %	Main mono-saccha-rides	Dominant taxa
Colonization (0–7 days)	11.3	3.4	Glu, Fuc, Rha	*Pseudomonadota (Pseudomonas)*	7.4	2.9	Man, Fuc, Glu	*Pseudomonadota (Undibacterium); Actinomycetota (Pseudarthrobacter)*
Matrix accumulation (7–15 days)	3.6	40.3	Rha, Rib, Fuc, Man	*Pseudomonadota (Pseudomonas)*	5.8	31.9	Rib, N-acGlcN, Xyl	*Pseudomonadota (Acidovorax, Pseudomonas); Actinomycetota (Arthrobacter, Pseudarthrobacter)*
Maturation and dispersion (15–40 days)	3.9	24.6	N-acGlcN, Fuc	*Pseudomonadota; Actinomycetota (Pseudarthrobacter)*	3.7	21.0	Fuc, Xyl, Rib, N-acGlcN	*Pseudomonadota (Undibacterium); Actinomycetota (Pseudarthrobacter)*
Re-colonization and biofilm restoration (70–100 days)	10.4	6.2	Xyl, GlcN, Fuc	*Pseudomonadota (Paucimonas, Brevundimonas, Pseudomonas); Bacillota (Desulfosporosinus)*	11.7	10.5	Xyl, GlcN, Fuc	*Pseudomonadota (Acidovorax); Actinomycetota (Pseudarthrobacter)*
NO_3^- reduction rate, mg/L/day	6.5				17.1			
**MW2 biofilm in RW contamination conditions**								
Cr^{6+}-reduction rate, mkg/(L·day)	270							
Tc^{7+}-reduction rate, mkg/(L·day)	660							
U^{6+}-reduction rate, mkg/(L·day)	1625							
